# Supplementary material for: Mobility restrictions for the control of epidemics: When do they work?
Source: PLoS One. 2020 Jul 6;15(7):e0235731. doi: 10.1371/journal.pone.0235731 (PMC7337314; doi:10.1371/journal.pone.0235731)
Supplement: S1 Appendix — (PDF) [file pone.0235731.s001.pdf]

# Mobility restrictions for the control of epidemics: When do they work?

Baltazar Espinoza[1*], Carlos Castillo-Chavez[2], Charles Perrings[3]

**1** Simon A. Levin Mathematical and Computational Modeling Sciences Center, Arizona State University, Tempe, Arizona, United States of America.
**2** Retired Professor, Arizona State University, Tempe, Arizona, United States of America.
**3** School of Life Sciences, Arizona State University, Tempe, Arizona, United States of America.

* Corresponding author
E-mail: baltazar.espinoza@asu.edu

## Supporting Information

### COVID-19 Basic Reproductive Number in Homogeneous Risk Communities

By following the next generation approach [36, 37], we computed the basic reproductive number

$$\mathcal{R}_0 = \beta\left(\frac{q}{\kappa} + \frac{l\alpha}{(\alpha+\gamma_1+\delta)(\gamma_2+\delta)} + \frac{1}{\alpha+\gamma_1+\delta}\right).$$

The basic reproductive number of model (1) gives the average number of secondary infections produced by a typical infectious individual during its infectious period $\left(\frac{\beta}{\alpha+\gamma_1+\delta}\right)$, the secondary cases generated by asymptomatic exposed individuals $\frac{q\beta}{\kappa}$ and, the secondary cases produced by the fraction of diagnosed individuals $\frac{\alpha}{(\alpha+\gamma_1+\delta)(\gamma_2+\delta)}$ at the reduced infectiousness $l\beta$.

### COVID-19 Dynamics in Heterogeneous Risk Communities

In heterogeneous communities, new cases of infection per unit time among the Community $j$ resident population are modeled by incorporating the *effective density* or *effective population size* - the expected amount of residents and visitors sojourning in each community at time $t$. Community $j$ residents can get infected at their community

of residency (with risk of infection $\beta_j$) or while spending time in Community $i$ (with risk of infection $\beta_i$), modeled as follows

$$\varphi(j) = \beta_i p_{ji} S_j \frac{p_{ii} I_i + p_{ji} I_j}{p_{ii} N_i + p_{ji} N_j} + \beta_j p_{jj} S_j \frac{p_{ij} I_i + p_{jj} I_j}{p_{ij} N_i + p_{ji} N_j}.$$

Since we are budgeting times of residency proportions, then $p_{ii} + p_{ij} = 1$. In the two communities system, we use $t_i$ to denote the Community $i$ residents' average proportion of time in Community $j$ while $1 - t_i$ denotes the average proportion of time that Community $i$ residents spend in their place of residency. Hence, the expected proportion of infected population sojourning in Community $i$ at time $t$ is expressed as follows:

$$\frac{(1-t_i)I_i + t_j I_j}{(1-t_i)N_i + t_j N_j}$$

The proposed model assumes that COVID-19-diagnosed cases do not travel across communities and these individuals are exclusively in contact with the local population. By using the single community model (1) as baseline model, the dynamics of COVID-19 on a two communities landscape, with distinct risk of infection, can be described by the following system of differential equations

$$\begin{cases} \dot{S}_i = & -(1-t_i)\beta_i S_i \left( \frac{(1-t_i)(I_i+qE_i)+t_j(I_j+qE_j)}{(1-t_i)N_i+t_jN_j} + \frac{lJ_i}{N_i} \right) \\ & -t_i \beta_j S_i \frac{t_i(I_i+qE_i)+(1-t_j)(I_j+qE_j)}{t_iN_i+(1-t_j)N_j} \\ \dot{E}_i = & (1-t_i)\beta_i S_i \left( \frac{(1-t_i)(I_i+qE_i)+t_j(I_j+qE_j)}{(1-t_i)N_i+t_jN_j} + \frac{lJ_i}{N_i} \right) \\ & +t_i \beta_j S_i \frac{t_i(I_i+qE_i)+(1-t_j)(I_j+qE_j)}{t_iN_i+(1-t_j)N_j} - \kappa E_i \\ \dot{I}_i = & \kappa E_i - (\alpha + \gamma_1 + \delta) I_i \\ \dot{J}_i = & \alpha I_i - (\gamma_2 + \delta) J_i \\ \dot{R}_i = & \gamma_1 I_i + \gamma_2 J_i \end{cases} \tag{2}$$

where $i, j \in \{1, 2\}$ and $i \neq j$.

## COVID-19 Basic Reproductive Number in Heterogeneous Risk Communities

System's (2) basic reproductive number is computed by following the next generation approach [36, 37]. Consider the infectious compartments $E_1$, $I_1$, $J_1$, $E_2$, $I_2$

and $J_2$, evaluating at the DFE, leads to $S_1(0) = N_1$ and $S_2(0) = N_2$. Then

$$\mathcal{F} = \begin{pmatrix} (1-t_1)\beta_1 S_1 \left(\frac{(1-t_1)(I_1+qE_1)+t_2(I_2+qE_2)}{(1-t_1)N_1+t_2N_2} + \frac{lJ_1}{N_1}\right) + t_1\beta_2 S_1 \frac{t_1(I_1+qE_1)+(1-t_2)(I_2+qE_2)}{t_1N_1+(1-t_2)N_2} \\ 0 \\ 0 \\ t_2\beta_1 S_2 \frac{(1-t_1)(I_1+qE_1)+t_2(I_2+qE_2)}{(1-t_1)N_1+t_2N_2} + (1-t_2)\beta_2 S_2 \left(\frac{t_1(I_1+qE_1)+(1-t_2)(I_2+qE_2)}{t_1N_1+(1-t_2)N_2} + \frac{lJ_2}{N_2}\right) \\ 0 \\ 0 \end{pmatrix}$$

and

$$\mathcal{V} = \begin{pmatrix} \kappa E_1 \\ -\kappa E_1 + (\alpha+\gamma_1+\delta)I_1 \\ -\alpha I_1 + (\gamma_2+\delta)J_1 \\ \kappa E_2 \\ -\kappa E_2 + (\alpha+\gamma_1+\delta)I_2 \\ -\alpha I_2 + (\gamma_2+\delta)J_2 \end{pmatrix}.$$

then, the basic reproductive number of model (2), in the presence of mobility, is given by the spectral radius of the next generation matrix $-FV^{-1}$. Note that the basic reproductive number is a function of the community-specific mobility, risk levels and community density, ($\mathbb{P}$, $\beta_i$ and $N_i$, respectively).

### COVID-19 Final Epidemic Size in Heterogeneous Risk Communities

We compute the community-specific final epidemic size, as function of residency times. By assuming $S_i(0) = N_i$, $E_i(0) = I_i(0) = J_i(0) = R_i(0) = 0$ and, by adding the first two equations in model (2), $\dot{S}_i + \dot{E}_i = -\kappa \hat{E}_i$, then $E_i^\infty = 0$ and $\hat{E}_i = (N_i - S_i^\infty)\frac{1}{\kappa}$, where $\hat{f}(t) = \int_0^\infty f(s)ds$ and $f^\infty = \lim_{t\to\infty} f(t)$. Following the same reasoning $\hat{I}_i = (N_i - S_i^\infty)\frac{1}{\alpha+\gamma_1+\delta}$ and, $\hat{J}_i = (N_i - S_i^\infty)\frac{\alpha}{(\alpha+\gamma_1+\delta)(\gamma_2+\delta)}$.

From model's (2) first equation, the secondary infections among the Community $i$,

produced by Community $i$ and Community $j$ individuals are given by

$$\begin{aligned}
\log\left(\frac{N_1}{S_1^\infty}\right) = \;& (N_1 - S_1^\infty)\left((1-t_1)\beta_1\left(\frac{(1-t_1)\left(\frac{1}{\alpha+\gamma_1+\delta}+\frac{q}{\kappa}\right)}{(1-t_1)N_1+t_2N_2} + \frac{\frac{\alpha l}{(\alpha+\gamma_1+\delta)(\gamma_2+\delta)}}{N_1}\right)\right.\\
& \left. + \, t_1\beta_2\frac{t_1\left(\frac{1}{\alpha+\gamma_1+\delta}+\frac{q}{\kappa}\right)}{t_1N_1+(1-t_2)N_2}\right)\\
& +(N_2 - S_2^\infty)\left((1-t_1)\beta_1\frac{t_2\left(\frac{1}{\alpha+\gamma_1+\delta}+\frac{q}{\kappa}\right)}{(1-t_1)N_1+t_2N_2} \;+\; t_1\beta_2\frac{(1-t_2)\left(\frac{1}{\alpha+\gamma_1+\delta}+\frac{q}{\kappa}\right)}{t_1N_1+(1-t_2)N_2}\right)\\
\log\left(\frac{N_2}{S_2^\infty}\right) = \;& (N_1 - S_1^\infty)\left((1-t_2)\beta_2\frac{t_1\left(\frac{1}{\alpha+\gamma_1+\delta}+\frac{q}{\kappa}\right)}{t_1N_1+(1-t_2)N_2} \;+\; t_2\beta_1\frac{(1-t_1)\left(\frac{1}{\alpha+\gamma_1+\delta}+\frac{q}{\kappa}\right)}{(1-t_1)N_1+t_2N_2}\right)\\
& +(N_2 - S_2^\infty)\left((1-t_2)\beta_2\left(\frac{(1-t_2)\left(\frac{1}{\alpha+\gamma_1+\delta}+\frac{q}{\kappa}\right)}{t_1N_1+(1-t_2)N_2} + \frac{\frac{\alpha l}{(\alpha+\gamma_1+\delta)(\gamma_2+\delta)}}{N_2}\right)\right.\\
& \left. + \, t_2\beta_1\frac{t_2\left(\frac{1}{\alpha+\gamma_1+\delta}+\frac{q}{\kappa}\right)}{(1-t_1)N_1+t_2N_2}\right)
\end{aligned}$$

which is expressed in vector form as

$$\begin{bmatrix} \log\left(\frac{N_1}{S_1^\infty}\right) \\ \\ \log\left(\frac{N_2}{S_2^\infty}\right) \end{bmatrix} = \begin{bmatrix} B_{11} & B_{12} \\ B_{21} & B_{22} \end{bmatrix} \begin{bmatrix} 1-\frac{S_1^\infty}{N_1} \\ \\ 1-\frac{S_2^\infty}{N_2} \end{bmatrix} \tag{3}$$

where

$$\begin{aligned}
B_{11} &= \left((1-t_1)\beta_1\left(\frac{(1-t_1)\left(\frac{1}{\alpha+\gamma_1+\delta}+\frac{q}{\kappa}\right)}{(1-t_1)N_1+t_2N_2} + \frac{\frac{\alpha l}{(\alpha+\gamma_1+\delta)(\gamma_2+\delta)}}{N_1}\right) \;+\; t_1\beta_2\frac{t_1\left(\frac{1}{\alpha+\gamma_1+\delta}+\frac{q}{\kappa}\right)}{t_1N_1+(1-t_2)N_2}\right)N_1,\\
B_{12} &= \left((1-t_1)\beta_1\frac{t_2\left(\frac{1}{\alpha+\gamma_1+\delta}+\frac{q}{\kappa}\right)}{(1-t_1)N_1+t_2N_2} \;+\; t_1\beta_2\frac{(1-t_2)\left(\frac{1}{\alpha+\gamma_1+\delta}+\frac{q}{\kappa}\right)}{t_1N_1+(1-t_2)N_2}\right)N_2,\\
B_{21} &= \left((1-t_2)\beta_2\frac{t_1\left(\frac{1}{\alpha+\gamma_1+\delta}+\frac{q}{\kappa}\right)}{t_1N_1+(1-t_2)N_2} \;+\; t_2\beta_1\frac{(1-t_1)\left(\frac{1}{\alpha+\gamma_1+\delta}+\frac{q}{\kappa}\right)}{(1-t_1)N_1+t_2N_2}\right)N_1,\\
B_{22} &= \left((1-t_2)\beta_2\left(\frac{(1-t_2)\left(\frac{1}{\alpha+\gamma_1+\delta}+\frac{q}{\kappa}\right)}{t_1N_1+(1-t_2)N_2} + \frac{\frac{\alpha l}{(\alpha+\gamma_1+\delta)(\gamma_2+\delta)}}{N_2}\right) + t_2\beta_1\frac{t_2\left(\frac{1}{\alpha+\gamma_1+\delta}+\frac{q}{\kappa}\right)}{(1-t_1)N_1+t_2N_2}\right)N_2.
\end{aligned} \tag{4}$$

Model's (2) final size relation is denoted by the community-specific final proportion of infected individuals (or attack rate).

The eigenvalues of the matrix $B$, on the final epidemic size expression (3), are the same as those of the next generation matrix. Therefore, the global - communities integrated - basic reproductive number is also the spectral radius of $B$, [26]. In addition, under this Lagrangian framework the global basic reproductive number is a function of the mobility matrix ($\mathbb{P}$) and the community-specific basic reproductive numbers ($\mathcal{R}_{0i}$), defined in the absence of mobility ($t_1 = t_2 = 0$), so that, $\mathcal{R}_0 = f(\mathbb{P}, \mathcal{R}_{01}, \mathcal{R}_{02})$.

Let $s_i^\infty = \lim_{t\to\infty} \frac{S_i(t)}{N_i}$, represent the proportion of the population remained susceptible

at the end of the epidemic. Then, from equation (3)

$$\begin{aligned} s_1^\infty &= \exp[-B_{11}(s_1^\infty - 1) - B_{12}(s_2^\infty - 1)], \\ s_2^\infty &= \exp[-B_{21}(s_1^\infty - 1) - B_{22}(s_2^\infty - 1)]. \end{aligned}$$

which in terms of the total number of cases from Community $i$ over the course of the epidemic ($y_i = 1 - s_i^\infty$), takes the form

$$\begin{aligned} y_1 &= 1 - \exp[-y_1 B_{11}] \exp[-y_2 B_{12}], \\ y_2 &= 1 - \exp[-y_1 B_{21}] \exp[-y_2 B_{22}]. \end{aligned} \tag{5}$$

System's (5) solution $(y_1^*, y_2^*)$, represents the community-specific attack rates. Therefore, the total final epidemic size ($\psi$) as a function of the community-specific density, risk and mobility levels, is given by

$$\psi(N_1, N_2, \mathcal{R}_{01}, \mathcal{R}_{02}, t_1, t_2) = N_1 y_1^* + N_2 y_2^*$$
