# Supplementary material for: Mobility restrictions for the control of epidemics: When do they work?
Source: PLoS One. 2020 Jul 6;15(7):e0235731. doi: 10.1371/journal.pone.0235731 (PMC7337314; doi:10.1371/journal.pone.0235731)
Supplement: Attachment — Submitted filename: PaperReview_tex.pdf [file pone.0235731.s002.pdf]

# Review of *Mobility restrictions for the control of epidemics: When do they work?* by Espinoza et. al.

May 10, 2020

Mobility restriction from/to the area impacted by an disease outbreak is one of the primary strategies that have been employed frequently to contain epidemics spatially and curtail the overall epidemic. In current manuscript use mathematical models to understand when do these mobility restrictions work. They do so by considering two adjoining populations with different characteristics: 1) High risk, High density, with inefficient medical resources (HRC) and 2) Low risk, Low density, with efficient resources (LRC). In their model, HRC and LRC are differentiated by different reproductive numbers (rate of infections) and both are connected by lagrangian movement. Authors find that their exist situation when mobility restriction can have counter productive effect when measured by overall epidemic size rather than area specific sizes. In particular, they find that as people from HRC area spend more and more time in LRC, it can reduce the overall epidemic size. This happens as R0 of two populations are different and more time people from higher R0 area spend in lower R0 area, it reduces the global R0 value. The methods employed in this manuscript to model movements between two locations have broader applicability as well as results obtained shed important light into how mobility restrictions work in general. Therefore, I would recommend accepting this manuscript. However, I have couple of minor points that I think authors can address to improve the manuscript:

1. The Lagrangian approach used for modeling the movement between two populations results in entire population of high risk community spending sometime in low risk community. This seems very unlikely and limits the applicability of their results. For example, authors discuss how at least across national boundaries, epidemics are addressed in a area-specific manner. While it is true, I do not think that their results shed any light on that in current form. Even when there is high mobility across two nations, only a proportion of population will travel and spend time.

2. It would be great for readers if authors give examples of situations where scenarios illustrated by authors in the manuscript can arise. For example, interactions between richer and poorer regions within a city, such that people from poorer region spend a lot of time in richer region for employment etc.
