# Supplementary material for: Mobility restrictions for the control of epidemics: When do they work?
Source: PLoS One. 2020 Jul 6;15(7):e0235731. doi: 10.1371/journal.pone.0235731 (PMC7337314; doi:10.1371/journal.pone.0235731)
Supplement: Attachment — Submitted filename: Response to Reviewers.pdf [file pone.0235731.s003.pdf]

We carefully reviewed each of the comments made by the reviewers and made edits to the manuscript. The comments and suggestions made by the reviewers addressed key aspects of our study, we feel that our manuscript is much improved now and we thank the reviewers for their helpful comments. We have addressed each point and made changes to our manuscript in blue. The reviewer's comments are addressed below.

- Comments from journal:

  1. Please ensure that your manuscript meets PLOS ONE's style requirements, including those for file naming.
     Response: Thank you. We made edits to the manuscript to ensure that it holds PLOS ONE's journal standards.
  2. Please upload a copy of Figures 1 to 4, to which you refer in your text. If the figures are no longer to be included as part of the submission please remove all reference to them within the text.
     Response: Thank you. We have edited all the figures according to the PLOS ONE's journal standards.

- Comments from reviewer #1:

  1. First, the figures need to be made according to the standards of PLOS one. For instance, some figures have no axis-labels and not tick labels. Please revise all graphs.
     Response: Thank you. We have edited all the figures according to the PLOS ONE's journal standards.
  2. Second, while the supporting information are not part of the main text, but they provide an important part of the manuscript and I find it replete of mathematical typos that makes it difficult to read and might affect the quality of the paper. Please revise it carefully.
     Response: Thank you for the comment. We have reviewed the formulas and fixed the typos in the SI Appendix. In addition we decided to move the section "Disease Dynamics in Homogeneous Risk Communities" to the body of the manuscript since we think the it complements the structure of the main text.
  3. While the results of the manuscript are computationally sound, I find the authors mention sanitation, trade and culture difference in many places in the manuscript while they are not explicitly incorporated in the model. Lack of their incorporation leads me to find that the results are not surprising to some degree. The reason is that the authors, assume that when an individual from one community spends some time in the other community, he/she will have the undergo the transmission rate of the new community. That is counter to the statement of the influence of personal sanitation (since it is face-to-face transmission) and culture influence as they will not change over small periods of time and if they do, they will be carried over to the other community. Thus, that would lead mathematically to a force of infection on those individuals and, for instance, smaller size of epidemics in the HRC's. More explicit modeling might be required to clear that up.
     Response: Thank you. We recognize human behavior as an important factor driving disease dynamics and, that it differs on distinct environments, modifying the local risk of infection on visitors. Although it is not explicitly formulated in the manuscript, community components - sanitation, trade and culture, income, education, health-care access, cultural practices, and so on - are assumed to produce differences in the community-specific risk of infection (β_i). Therefore, impacting the community-specific disease dynamics and, in the presence of mobility, the global disease dynamics and the final epidemic

size. In other words, we recognize the risk of infection is highly affected by the community-specific characteristics. For instance, we assume that an individual visiting the New York's neighborhoods of Queens or Brooklyn, experiences a high COVID-19 risk of infection during the visiting time. The proposed model takes in account this former component, envisioning a likelihood of infection tied to the community-specific characteristics. For clarification, we included in the manuscript the following sentence: "We recognize that human behavior is an important determinant of disease dynamics, and that behavior differs across environments, either ameliorating or exacerbating the impact of the local risk of infection on visitors. Moreover, we also recognize that the risk of infection is strongly influenced by community-specific characteristics that alter local and global disease dynamics. It is assumed that the community-specific infection risk reflects community attributes that include income, education, health-care access, cultural practices, and so on." Lines 121-127.

- Comments from reviewer #2:

  1. The Lagrangian approach used for modeling the movement between two populations results in entire population of high-risk community spending sometime in low risk community. This seems very unlikely and limits the applicability of their results. For example, authors discuss how at least across national boundaries, epidemics are addressed in a area-specific manner. While it is true, I do not think that their results shed any light on that in current form. Even when there is high mobility across two nations, only a proportion of population will travel and spend time.
     Response: Thank you for pointing this out. We agree our results hold whenever the traveling population size is comparable to the community population size. Moreover, we agree that such an scenario is very unlikely to occur at the population size of countries. Consequently, we added some lines to clarify that our results are limited to smaller geographical scales. In order to address this comment we replaced the lines discussing about how epidemics are addressed across national boundaries with the following : "The evidence suggests that COVID-19 is overwhelmingly being addressed from the perspective of area-specific risk. Disease control measures are aimed less at reducing the final epidemic size than at containing the disease in particular areas. Our results hold at scales where the traveling population size is comparable to the community population - we address disease dynamics exhibited at the scale of inter-community transmission." (Lines 293-297) and, "Internationally, however, it is clear that disease control is aimed at the final epidemic size within and not across nation states. In such cases, while the use of mobility restrictions to control disease in particular jurisdictions might increase the overall final epidemic size, it can still lower the country-specific final epidemic size." (Lines 306-310).

  2. It would be great for readers if authors give examples of situations where scenarios illustrated by authors in the manuscript can arise. For example, interactions between richer and poorer regions within a city, such that people from poorer region spend a lot of time in richer region for employment etc.
     Response: Thank you for suggest this. To complement our manuscript addressing this comment, we have added the following lines: " Within individual countries there are examples of regions governed by a single health authority but including dramatic differences in living conditions. The Brazilian communities in Rio de Janeiro also known as the "favelas"; the Primrose area neighboring the Makause settlement in Johannesburg, South Africa; the slum populations of Mumbai, India; the Santa Fe neighborhood in Mexico City, Mexico; and the New York neighborhoods of Queens, Brooklyn and Manhattan in the USA are all examples of areas where dramatic differences in living conditions coexist within

a single public health area. In such cases, disease control might be motivated by the final epidemic size across communities." (Lines 298-306).
